# Aerobic activity significantly reduces blood pressure among hypertensive adults in Africa: a systematic review and meta-analysis

**DOI:** 10.3389/fpubh.2025.1548584

**Published:** 2025-05-19

**Authors:** Titus Robert Leeyio, Nicholas Philip Katto, Faiza Mohamed Juma, Farida Iddy Mkassy, Anthony Cuthbert Liwa, Sarah Shali Matuja, Daniel Byamungu, Philip Ayieko, Deogratius Bintabara, Eveline Thobias Konje

**Affiliations:** ^1^Department of Epidemiology and Biostatistics, School of Public Health, Catholic University of Health and Allied Sciences, Mwanza, Tanzania; ^2^Department of Pharmacology, Weill School of Medicine, Catholic University of Health and Allied Sciences, Mwanza, Tanzania; ^3^Department of Internal Medicine, Weill School of Medicine, Catholic University of Health and Allied Sciences, Mwanza, Tanzania; ^4^Department of Physiology, Weill School of Medicine, Catholic University of Health and Allied Sciences, Mwanza, Tanzania; ^5^Mwanza Intervention Trial Unit, Mwanza, Tanzania; ^6^Department of Community Medicine, The University of Dodoma, Dodoma, Tanzania

**Keywords:** aerobic activity, physical exercise, hypertension, blood pressure, hypertensive patients, Africa

## Abstract

**Background:**

Sub-Saharan Africa is faced with the increasing prevalence of high blood pressure, with projections estimating that 216.8 million people will be affected by 2030. Aerobic activity has been recognized for its cardiovascular benefits. However, its effect remains under-explored, and the findings are largely inconsistent. Therefore, this review aimed to estimate the overall effect of aerobic activity on blood pressure among hypertensive adults receiving medication in Africa.

**Methods:**

Randomized clinical trials conducted in Africa and published between 2000 and September 2024 were considered eligible if they included hypertensive adults aged 18 years and older. Studies were identified through searches on PubMed, African Journal Online, Hinari, and Science Direct databases. In addition, the Cochrane risk-of-bias tool (ROBIS-2) was used for quality assessment.

**Results:**

Of the 683 articles identified, eight qualified for qualitative assessment, comprising 1,112 participants on antihypertensive medication (625 in the intervention group and 487 in the control group). The follow-up duration ranged from 6 to 16 weeks, with different aerobic modalities such as brisk walking, dance, and bicycle ergometer. These activities were performed either continuously or at different time intervals (three times per week), lasting between 30 and 60 min. The overall risk of bias was moderate, and the intensity of the training was based on VO_2_Max (64–79%) and VO_2_Peak (40–79%). The main findings suggest a significant pooled reduction in systolic blood pressure, with a mean difference (MD) of 5.40 mmHg [95% CI: −9.05 to −1.75], and a modest reduction in diastolic blood pressure, with an MD of 1.90 mmHg [95% CI: −3.81 – 0.01]. Subgroup analysis revealed that interventions lasting for at least 8 weeks produced stronger effects than those implemented for more than 8 weeks.

**Conclusion:**

Our finding shows that adherence to antihypertensive medications in addition to aerobic activity effectively reduced blood pressure among hypertensive adults in Africa, particularly at 8 weeks, with a slightly diminished effect in prolonged durations. It is, therefore, imperative to conduct further studies in this area of hypertension management.

**Systematic review registration:**

https://www.crd.york.ac.uk/PROSPERO/view/CRD42024614250.

## 1 Introduction

Hypertension is a leading cause of morbidity and mortality, affecting 1.28 billion adults worldwide, with 25% of men and 20% of women impacted by cardiovascular diseases (CVDs) (1). CVDs remain the foremost cause of global premature deaths, accounting for 30% of all deaths and affecting between 31 and 38% of adults (1). The prevalence of hypertension in Africa is projected to reach 54% among adults, making it the highest rate globally (2, 3). Alarmingly, only 7% of hypertensive adults have their blood pressure under control, with 93% of individuals at risk of complications such as stroke and heart failure (4). The prevalence of complications related to hypertensive disease ranges from 7 to 10% of adult hospital admissions in Africa, with heart failure accounting for 3–7% of these cases. Specifically, hypertension is responsible for 45% of deaths due to heart disease and 51% of deaths due to stroke (5). In 2019, approximately 26% of deaths in North Africa resulted from high blood pressure, and it is also the fourth leading risk factor in sub-Saharan Africa (SSA), accounting for 8.8% of deaths and over 1 million CVD deaths (4). Between 2000 and 2010, there was a 41% increase in the prevalence of high blood pressure, with projections estimating 216.8 million cases by 2030 (6). Currently, the global prevalence of hypertension is estimated at 54%, with less than half of adults (42%) diagnosed and treated. Furthermore, the prevalence of hypertension is estimated to range between 42 and 54% in SSA (7). The SPRINT trial demonstrated that the intensive control of blood pressure significantly reduces the risk of cardiovascular events and mortality among adults (8).

In addition to the well-established pharmacological interventions to prevent the undesirable effects of hypertension, non-pharmacological interventions, such as aerobic activity, healthy diets, weight management, reducing alcohol consumption, and smoking cessation, have been reported to significantly reduce the risk of cardiovascular diseases among hypertensive populations (9). Globally, the World Health Organization (2020) and the American Heart Association (2021) recommend that adults engage in at least 150–300 min per week of moderate-intensity aerobic activity or 75–150 min per week of vigorous-intensity aerobic activity, in addition to muscle-strengthening activities on 2 or more days per week. Activities such as brisk walking or cycling are encouraged to promote a healthy heart and prevent hypertension complications (American Heart Association, 2024, WHO, 2020). Adherence to these practices is low and inconsistent with <30% of adults in some African countries engaging in non-pharmacological interventions either continuously or regularly (10). Inconsistency in treatment adherence across African populations could be attributed to differences in cultural practices, personal exposure status, and differing national campaigns (11, 12). In high-income countries, different studies have established the effectiveness of aerobic activity in lowering the mean systolic and mean diastolic blood pressure, which is reported to reduce the risk of cardiovascular disease (11, 12).

However, inconsistent findings were reported from studies conducted in African countries. For instance, Cleven et al. (13) found no significant association between physical activity and high blood pressure reduction (13–15), while Saco-Ledo et al. (16) observed a significant association (16, 17). This inconsistency highlights a critical gap in the adoption of aerobic activity interventions in African settings. Hence, this study aimed to estimate the effect size of aerobic activity, compared to no intervention, in lowering blood pressure among African hypertensive adults.

## 2 Methods

A systematic review and meta-analysis of randomized clinical trials were conducted to estimate the effect size of aerobic activity in controlling hypertension among patients using antihypertensive medication. The population under study was hypertensive adults aged above 18 years, where the intervention was aerobic activity. The comparison group was hypertensive adults under medication. The outcome was a significant reduction in blood pressure. This study adhered to the PRISMA items and was registered on the PROSPERO platform with registration number CRD42024614250.

### 2.1 Search strategies

Four electronic databases, namely, PubMed, African Journal Online, Science Direct, and Hinari, were used for the literature search, focusing on randomized clinical trials conducted in Africa between 2010 and 2024. The search was carried out in December 2024, with keywords for the outcome of interest being “Hypertension,” OR “High Blood pressure,” OR “Systolic Blood pressure,” OR “Diastolic Blood pressure,” OR “Elevated Blood pressure.” The exposure terms included “Physical Activity,” “Aerobic,” “Regular Activity,” OR “Exercise,” OR “Physical Exercise,” combined with specific study designs and locations such as AND “community-based,” OR “randomized controlled trials,” AND “Africa.” In addition, a search for gray literature and unpublished studies in institutional repositories, conference proceedings, and organizational websites found no relevant studies for inclusion.

### 2.2 Eligibility criteria

In this study, we included African-based randomized control trials published in English between 2010 and 2024 with a definite diagnosis criterion for blood pressure (SBP>=140mmHg and DBP>=90mmHg) (18), or involving participants on medications for high blood pressure. Further studies that quantify aerobic activity in terms of type, frequency, and duration with a study population of adults aged 18 + years were considered eligible. Reviews, reports, letters, comments, and studies that did not provide specific measures of aerobic activity and studies that did not report the primary or secondary outcomes as related to high blood pressure reduction were excluded.

### 2.3 Study selection and data extraction

Initial study selection involved title and abstract screening using Rayyan online software, conducted by two independent researchers T.R.L. and N.P.K. Subsequent comparison was done by the same researchers. An independent researcher, F.M.J., was responsible for settling disagreements. Data extraction was carried out independently by the same two researchers, who later compared their results to avoid any errors in the presence of the third researcher. Any data extraction discrepancies were discussed and unresolved conflicts were adjudicated by F.M.J. MS Excel was tailored to extract data from the included studies, with key variables including age, sex, body mass index (BMI), volume of oxygen (V0_2_Max), use of medication, training interval, duration of aerobic activity (in minutes), frequency, and follow-up time.

Data on outcome variables were extracted by focusing on SBP and DBP with mean difference (MD) and 95% confidence intervals (CI) for both the control and the intervention groups. The control group comprised hypertensive participants who received medication, while the intervention group comprised adults who were subjected to aerobic activity or both aerobic activity and adherence to prescribed medication. The Cochrane risk-of-bias tool (19) was used to assess the quality of included studies, focusing on key domains: randomization processes, assignment to intervention, adherence to intervention, bias due to missing outcome data, measurement of the outcome, and selection of the reported result. The risk of bias was classified as high when methodological criteria were either not reported or not performed, as low when the criteria were properly implemented, and as having some concerns when the criteria were inadequately described, making it impossible to classify the risk as high or low.

### 2.4 Data analysis

In all studies included, V0_2_ Max and BP (SBP and DBP) were measured as continuous variables. The mean difference (MD) for both SBP and DBP was calculated for both the intervention and control groups. The effect size was calculated using the random-effects model and reported as MD with a 95% CI. The analysis results are presented as MD with a 95% CI. Statistical heterogeneity was assessed using the *I^2^* test, and inconsistency was considered significant at *I^2^* > 50%. The random-effects meta-analysis would be performed in case of unexplained heterogeneity in the studies. In addition, a *p* < 0.01 from Cochran’s *Q* test was deemed significant for heterogeneity (20). Subgroup analyses were conducted based on follow-up time. There was not enough information from the studies to perform subgroup analyses on the type of aerobic activity, the nature of conducting aerobic activity, and the intensity of intervention. Forest plots were used to display the mean difference of SBP, the outcome from synthesized studies, and the 95% CI; statistical significance was considered at a *p* < 0.05. Meta-analysis was performed using R software version 4.3.2.

## 3 Results

### 3.1 Search results

**Figure 1:**
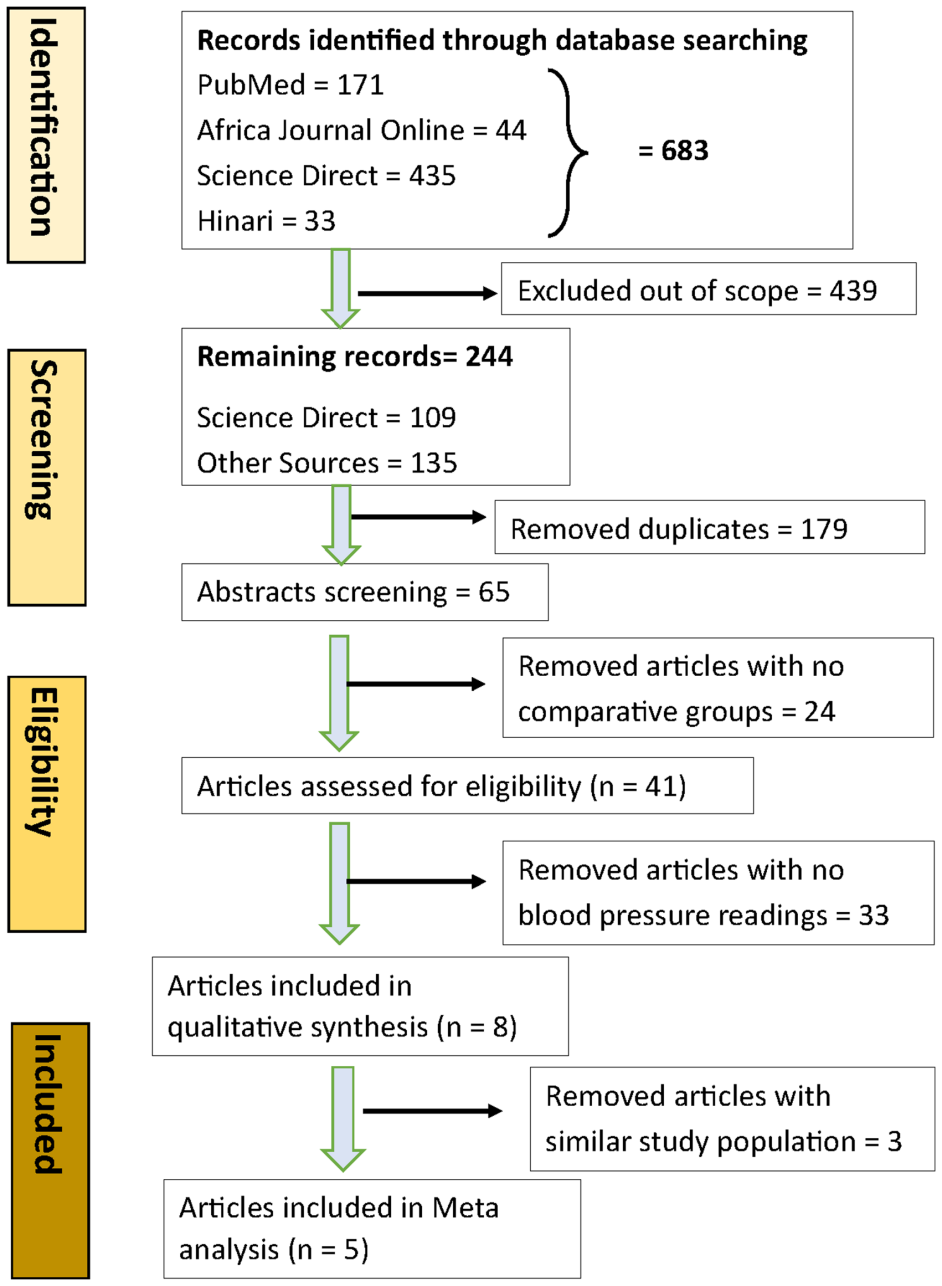
PRISMA flow chart.

Based on the search words across pre-identified databases, 683 randomized clinical trials were identified, of which several were excluded for different reasons: 439 studies were out of scope, 179 were duplicates, and 24 lacked a comparative group (see details in Figure 1). Only eight studies were included in the full-text article assessment (21–28). Of the eight studies, three were further excluded: one did not report mean difference for both the intervention and control groups and two used the same population at different times to achieve the same results. Therefore, only five studies were included in the meta-analysis (21, 22, 26–28).

### 3.2 Characteristics of studies

Of the eight included studies in Table 1 below, one was from Namibia, two were from Ethiopia, and five were from Nigeria. A total of 1,112 participants from the eight studies were analyzed. Of these, 625 participants were allocated to the intervention group, while 487 constituted the control group. All studies focused on adult populations; however, one study exclusively analyzed female participants, another included both male and female participants, and the remaining studies focused solely on male participants. In these studies, all participants were using methyldopa antihypertensive drugs. All studies used randomization with three studies reporting double blinding. Lamina et al. (23) reported blinding participants to their assignment groups, and placebo tablets were given during the washout period to mimic antihypertensive medication. In addition, Lamina et al. (24) implemented a washout period during which participants received a placebo to maintain blinding to the effects of their previous medication.

**Table 1:** Study characteristics.

References	Country	Sample Size	Sample group	Average age (years)	Sex of participants	Intervention	Follow-up time	Outcome results	VO_2_ Max	% Adherence to medication
Lamina et al. (23)	Nigeria	357	I (interval):140, C (Continuous): 112	I: 58.63 ± 7.22	Male	Moderate-intensity cycling on an ergometer	8 weeks	SBP: −12.8 mmHg, DBP: −7.8 mmHg	I: 37.46 ± 7.45	NR
			C: 105	C: 58.27 ± 6.24					C: 22.82 ± 7.44	
Lamina et al. (26)	Nigeria	245	I: 140	I: 58.40 ± 6.91	Male	Moderate-intensity cycling on an ergometer	8 weeks	SBP: −10.3 mmHg, DBP: −6.9 mmHg	I: 37.46 ± 7.42	NR
			C: 105	C:58.27 ± 6.24					C: 22.82 ± 7.44	
Lamina et al. (25)	Nigeria	217	I: 112	I:58.63 ± 7.22	Male	Continuous aerobic training (60–70% of HR max) – Bicycle ergometer	8 weeks	SBP: −9.6 mmHg, DBP: −6.5 mmHg	I: 20.69 ± 12.49	NR
			C: 105	C:58.27 ± 6.24					C:21.23 ± 5.76	
Maruf et al. (28)	Nigeria	63	I: 30	I: 50.38 ± 8.4	Males and Females	Aerobic dance combined with antihypertensive drug therapy	12 weeks	SBP: −8.6 mmHg, DBP: −5.3 mmHg	NR	I:76.97% ± 16.5% C:73.3% ± 27.3%
			C: 33	C: 55.3 ± 8.1						
Latosik et al. (27)	Namibia	25	I: 15	NR	Female	10–12 min warm-up, 45 min Nordic walking and 10 min stretching	8 weeks	SBP: −8.5 mmHg	I: 28.8 (25.4,30.4)	NR
									C:29.4 (24.2,31.4)	
Lamina et al. (24)	Nigeria	245	I: 140	I:58.40 ± 6.91	Male	Interval exercise training, 3 times a week	12 weeks	SBP: −11.7 mmHg, DBP: −7.4 mmHg	I: 23.62 ± 9.15	100% Adherence
			C: 105	C: 58.27 ± 6.24					C: 21.23 ± 5.76	
Daimo et al. (22)	Ethiopia	24	I: 12	I: 38.42 ± 4.69	Male	Brisk walking, 3 days per week	16 weeks	SBP: −7.1 mmHg, DBP: −5.6 mmHg	NR	NR
			C: 12	C: 37.6 ± 3.60						
Alemayehu et al. (21)	Ethiopia	48	I: 36	I:45.64 ± 6.58	Male	Aerobic Dance and resistance exercise (standing planter flexion and squats)	12 weeks	SBP: −17.75 mmHg, DBP: −12.5 mmHg	I: 28.45 ± 4.85	NR
			C: 12	C: 47.08 ± 7.88					C: 4.92 ± 28.75	

NR, not reported; SBP, systolic blood pressure; DBP, diastolic blood pressure.

Finally, Lamina et al. (26) carried out double blinding, i.e., participants and assessors were blinded during the washout period by replacing medication with placebo tablets. Three studies reported participants’ adherence to pre-assigned medication; Maruf et al. (28) measured adherence to antihypertensive medication by counting the number of pills consumed in both the exercise and control groups. Participants were provided with their medications (Normoretic and Amlovar) every 3 weeks, with unused pills carefully counted to assess adherence. Both groups were monitored with the intervention group showing a 79.97% adherence rate, while the control group showed a 73.33% adherence rate (28).

Phone reminders and follow-up calls were also used to encourage participants to maintain both medication and aerobic activity adherence. In contrast, participants who were on antihypertensive medications, as reported by Lamina et al. (26), were asked to stop all the medication for a “washout period” before beginning the intervention. This cessation was carried out to ensure that any observed effects were due solely to aerobic activity and not due to previously taken medication (26). Furthermore, the washout period for this study did not influence the overall results in the meta-analysis.

### 3.3 Characteristics of the intervention

The reviewed studies reported follow-up durations ranging from 8 to 16 weeks, with four studies following up at 8 weeks, three at 12 weeks, and one at 16 weeks (21–28). Training sessions lasted 30–60 min, except for one study that did not report session duration. The interventions included various aerobic activities, such as brisk walking, resistance training, bicycle ergometer, and aerobic dance, with one group applying interval training for 15–20 min per session, three times a week over 12 weeks, at an intensity of 64–76% HRmax. Another group implemented continuous training with combined aerobic and resistance exercises, each lasting 30 min per session, at 64–70% VO_2_ max, three times weekly for 12 weeks.

Across studies, aerobic training was the primary intervention, with outcomes measured through changes in SBP, DBP, VO_2_Max, and, in some cases, lipid profiles and serum uric acid (SUA) levels before and after the intervention. Regarding intensity prescription, four studies used HRmax, two used VO2Max, and two did not report. Adherence to interventions was reported in two studies (76.97 and 69.1%), while six studies did not report adherence. These findings highlight the diversity in follow-up durations, training protocols, and intensity measures, emphasizing the focus on aerobic activity as a key intervention (see Table 2 below).

**Table 2:** Characteristics of the intervention.

References	Modality	Intervention period	Method	Session duration	Weekly frequency	Intensity	Outcome
Lamina et al. (23)	Bicycle ergometer (aerobic)	8 weeks	Interval Continuous	B:45 min F:60 min B:45 min F:60 min	3 times	60–79% HRmax 60–79% HRmax	Significant reduction in SBP
Lamina et al. (26)	Bicycle ergometer (aerobic)	8 weeks	Continuous	B:45 min F:60 min	3 times	60–79% HRmax 60% - 79 HRmax	Significant reduction in SBP
Lamina et al. (25)	Bicycle ergometer (aerobic)	8 weeks	Continuous	45 min-60 min	3 times	60–70% HRmax	Significant reduction in SBP & SUA Improvement in VO_2_ Max and psychosocial status
Maruf et al. (28)	Aerobic dance	12 weeks	Continuous	45 min	3 times	50–70% VO2max	Significant blood pressure control
Latosik et al. (27)	Nordic walking (aerobic)	8 weeks	Interval	45 min	NR	VO2max 40–60% 45–68% 38–69%	Decrease in SBP
Lamina et al. (24)	Bicycle ergometer (aerobic)	8 weeks	Interval	45 min-60 min	3 times	60–79% HRmax	Significant reduction in SBP, a significant increase in VO_2_ Max
Daimo et al. (22)	Brisk walking (aerobic)	16 weeks	Continuous	NR	3 times	NR	Reduction in SBP
Alemayehu et al. (21)	Aerobic dance Resistance intervention Combined	12 weeks	Continuous Interval Continuous	60 min 15–20 s 30 min	NR	64–76% HRmax	Significant reduction in BW, BMI, SBP, and DBP

NR, not reported; B, before; F, after; BMI, body mass index; SUA, serum uric acid; VO_2_ max, maximum oxygen consumption; HR, heart rate; SBP, systolic blood pressure; DBP, diastolic blood pressure.

### 3.4 Reduction of blood pressure (SBP and DBP) within and across the groups

Pooled results showed the effectiveness of the aerobic activity intervention in lowering blood pressure, with a − 14.03 mmHg reduction in SBP and an −8.04 mmHg reduction in DBP, with a *p*-value of <0.05. Similarly, individual studies showed varying results across different exercise types. For instance, Alemayehu et al. (21) and Latosik et al. (27) reported significant blood pressure reduction in SBP and DBP following aerobic dance, resistance exercise, bicycle ergometer, and brisk walking. Alemayehu et al. (21) observed a significant reduction in SBP and DBP of −9.36 mmHg and −6.64 mmHg, respectively, both with a *p* < 0.001. At the same time, Latosik et al. (27) reported that there was some reduction in SBP and DBP in the exercise group, with changes of −10.2 mmHg and −2.0 mmHg, respectively, compared to the control group, although these differences were not statistically significant.

Five studies reported a significant difference between the control and exercise groups in both SBP and DBP (21, 23, 24, 26, 27). One study showed a significant difference in SBP only among women (27), another study showed no significant difference in either group (28), and one study did not report its findings.

### 3.5 Analysis of the risk of Bias

**Figure 2:**
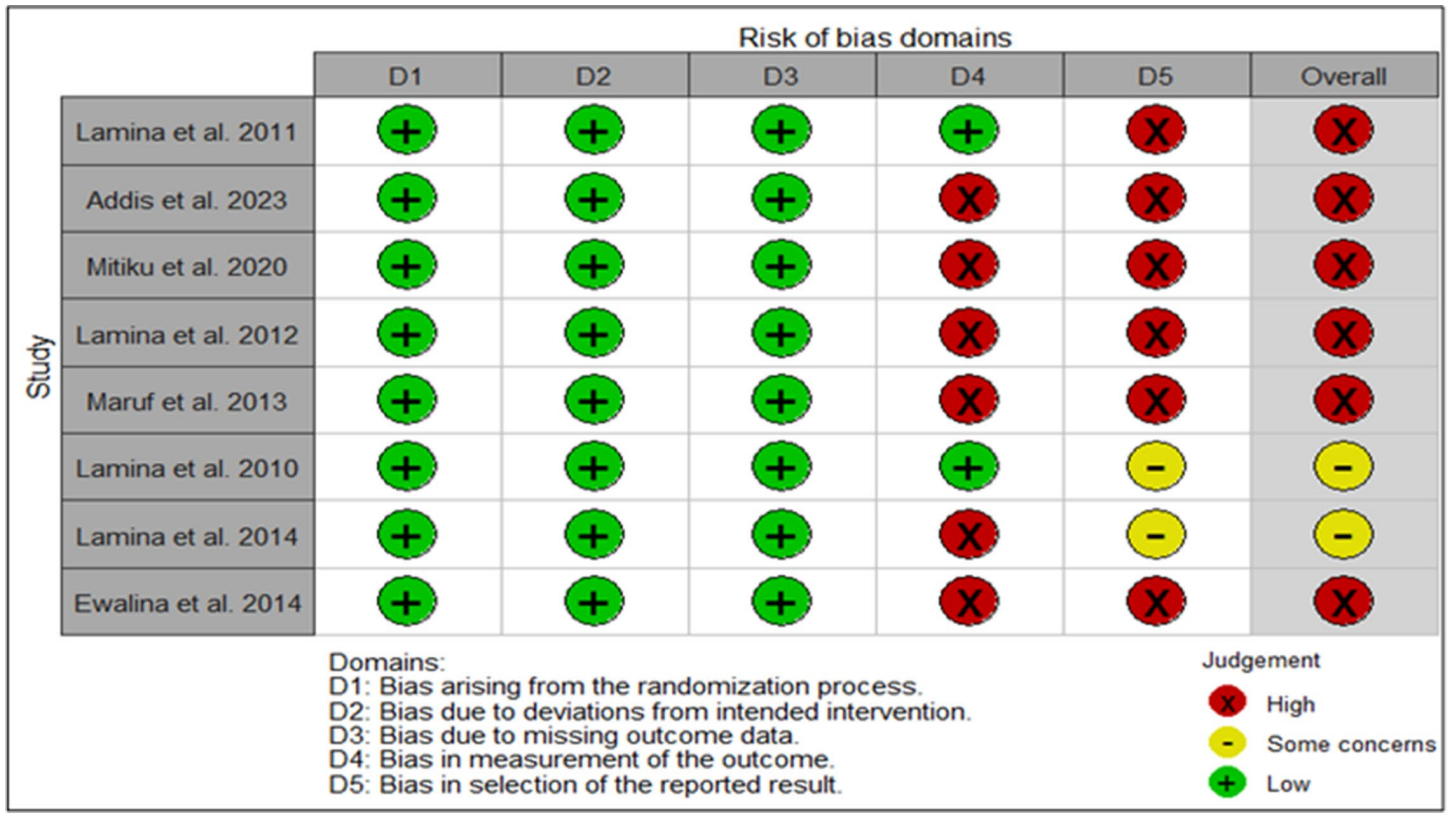
Risk of bias traffic plot.

The risk of bias in the included studies indicates a generally low risk in the randomization process (D1) and deviations from the intended interventions (D2) across all studies (see Figure 2). However, there is a notable high risk of bias in the measurement of outcomes (D4) for five studies and in the selection of reported results (D5) for six studies, with only two studies showing some concerns in these areas. In addition, two studies had some concerns regarding missing outcome data (D3). Overall, all included studies were judged to have a high risk of bias due to persistent issues in outcome measurement and reporting, despite demonstrating strong randomization and adherence to intervention.

### 3.6 Individual study results on diastolic and systolic blood pressure

The overall analysis of the included studies demonstrates variability in the effects of interventions on both DBP and SBP. Reductions in DBP ranged from minimal changes (−0.13 [95% CI: −5.42–5.16]) to more substantial decreases (−5.15 [95% CI: −7.03 to −3.27]), while reductions in SBP ranged from negligible effects (0.01 [−3.34–3.36]) to notable improvements (−10.87 [95% CI: −14.84 to −6.90]). However, not all studies showed significant effects, with some reporting CIs crossing zero, indicating uncertain or no meaningful impact. The weight of individual studies in the analysis further highlights these differences, with contributions ranging from 9.2 to 28.2% for DBP and 16.9 to 23.6% for SBP. These findings suggest that, while exercise-based interventions can significantly reduce blood pressure, the magnitude and consistency of the effects may vary, likely influenced by study precision, intervention protocols, and population characteristics (Table 3).

**Table 3:** Individual study results.

Study	DBP MD (95% CI)	Weight (random)	SBP MD 2 (95% CI)	Weight 2 (random)
Lamina et al. (24)	−1.12 [−2.10 to −0.14]	28.2%	−10.87 [−14.84 to −6.90]	20.4%
Alemayehu et al. (21)	−0.22 [−4.22–3.78]	13.1%	0.01 [−3.34–3.36]	21.9%
Daimo et al. (22)	−5.15 [−7.03 to −3.27]	23.5%	−6.50 [−9.08 to −3.92]	23.6%
Lamina et al. (25)	−1.27 [−2.70–0.16]	26.0%	−5.65 [−10.91 to −0.39]	17.2%
Maruf et al. (28)	−0.13 [−5.42–5.16]	9.2%	−4.01 [−9.41–1.39]	16.9%

### 3.7 Effectiveness of aerobic activity in diastolic blood pressure reduction and subgroup analysis

Overall aerobic activity is associated with a reduction in DBP, and the random-effects model presents a slightly larger but less conclusive effect size (MD) = −1.90 [95% C.I: −3.81 – 0.01] due to a borderline confidence interval. Significant heterogeneity among the five studies (*I^2^* = 74%) suggests variability in the effect sizes, which is reflected in the differing weights assigned to each study. The presence of significant heterogeneity, further confirmed by a low *p*-value (*p* < 0.01), implies that the studies do not all estimate the same true effect size on DBP reduction and that the observed effect could be due to chance alone.

**Figure 3:**
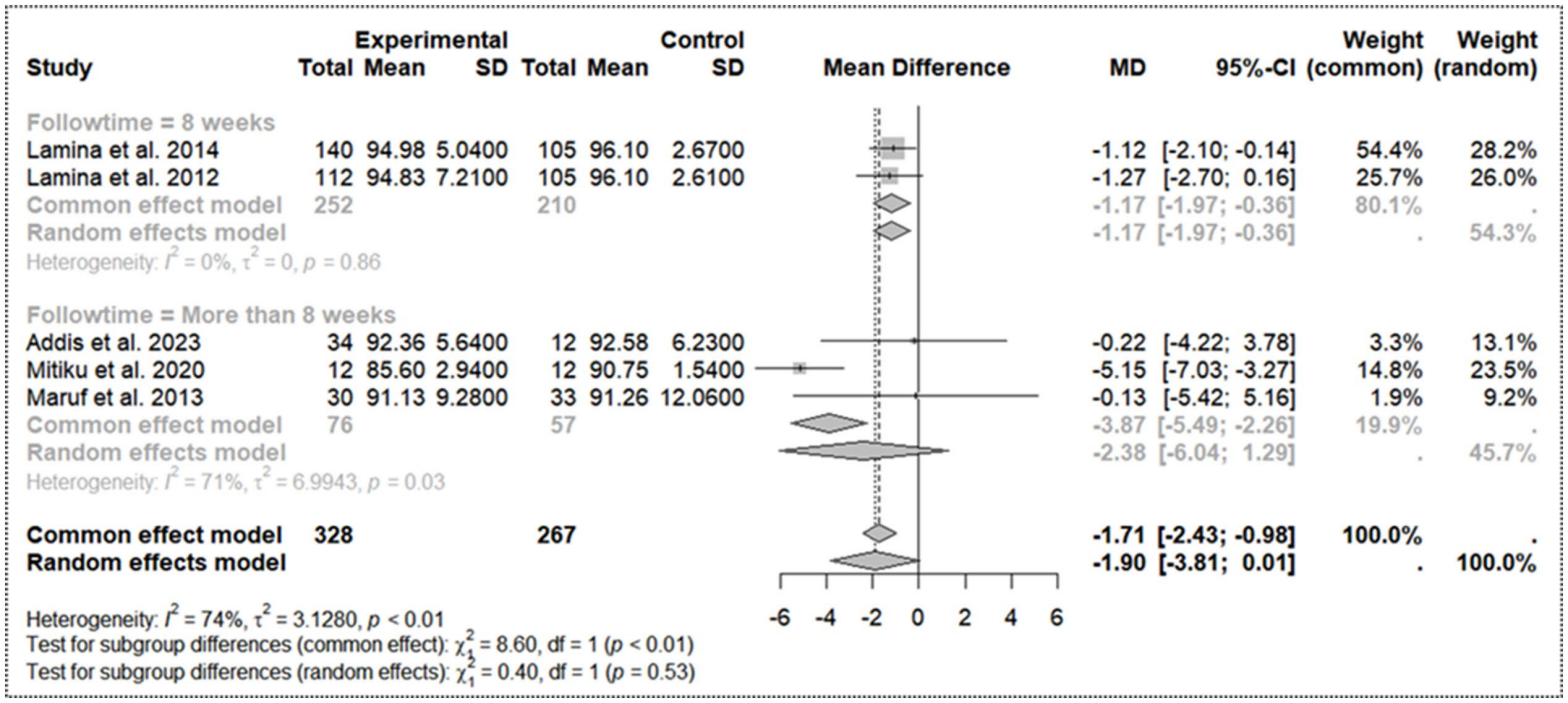
Sub group analysis on follow-up time for diastolic blood pressure.

Subgroup analysis (Figure 3) based on follow-up time shows differing effect sizes between studies with follow-up times of 8 weeks and those with more than 8 weeks. For the subgroup analysis with a follow-up time of 8 weeks, the MD is −1.17 [95% CI: −1.97 to −0.36], indicating a modest but significant effect, with no observed heterogeneity (*τ^2^* = 0). In contrast, the subgroup with follow-up times >8 weeks has a non-significant effect on the effectiveness of aerobic activity in DBP reduction MD = −2.38 [95% CI: −6.04 – 1.29] with substantial heterogeneity (*τ^2^* = 6.99). The test for subgroup differences is not statistically significant (*Q* = 0.40, d. f. = 1, *p* = 0.53). This finding suggests that, while the effect on DBP reduction appears more consistent and significant in the shorter period of the 8-week follow-up group, the variability in the follow-up group of more than 8 weeks leads to inconclusive differences between the subgroups.

### 3.8 Effectiveness of aerobic activity in systolic blood pressure reduction and subgroup analysis

A pooled analysis for the random-effects model is −5.40 mmHg [95% CI: −9.05 – -1.75]. However, synthesis of the studies reveals substantial heterogeneity (*I^2^* = 78%, *τ^2^* = 12.95, *p* < 0.01), indicating variability in the effect sizes across studies. The majority of studies reported a decrease in SBP in the experimental group, with Lamina et al. (24) and Daimo et al. (22) showing the largest reductions. Overall, the evidence suggests that the intervention leads to a clinically significant moderate reduction in SBP, ranging from 5 to 10 mmHg.

**Figure 4:**
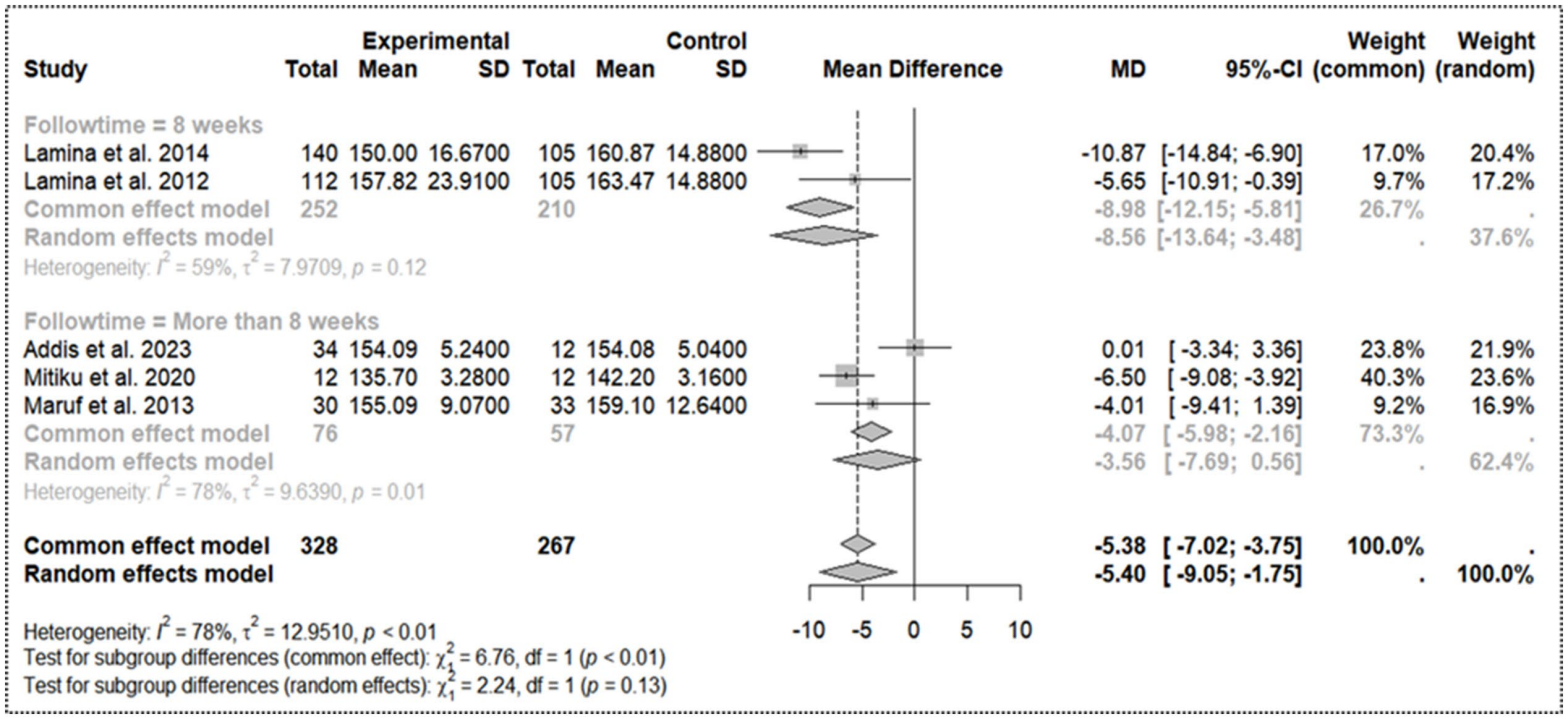
Sub group analysis on follow-up time for systolic blood pressure.

In the subgroup analysis (Figure 4) with follow-up times of 8 weeks, the pooled mean difference (MD) for SBP reduction is −8.56 [95% CI: −13.64 to −3.48], with substantial heterogeneity (*τ^2^* = 7.97). This difference indicates a significant and strong negative effect on the effectiveness of aerobic activity in SBP reduction. In the subgroup analysis with a follow-up of more than 8 weeks, the pooled MD is −3.56 [95% CI: −7.69 – 0.56], with even higher heterogeneity (*τ^2^* = 9.64), and the effect is not statistically significant. The test for subgroup differences is also non-significant (*Q* = 2.24, d. f. = 1, *p* = 0.1347), although a stronger effect is observed with shorter follow-up times.

## 4 Discussion

All studies included were relevant, valid, and evidence-based in answering the research question and context-specific to the African population. The significant pooled reduction in SBP (−5.4 mmHg) observed is consistent with other systematic reviews, which indicate that aerobic activity typically results in a reduction of 5–7 mmHg (29, 30), showing that aerobic activity reduces SBP across various populations, with the strongest effects observed in hypertensive adults (29). Although there was a significant reduction in blood pressure following aerobic physical exercise, these findings are similar to the findings found elsewhere, which reported a higher reduction rate of SBP after aerobic exercise. The mean reduction in SBP (up to −8.56 mmHg) was more substantial compared to DBP (up to −2.38 mmHg). Furthermore, the results of this study are in line with conclusions drawn by a meta-analysis conducted between 2020 and 2024 (31, 32).

Accounting for the duration of physical activity, a shorter duration of 8 weeks or less showed an improved reduction in both systolic and diastolic blood pressure. However, the presence of substantial heterogeneity (*I^2^* = 78%) indicates that the effects vary considerably across studies, likely due to differences in study populations, interventions, or methodologies. These findings are consistent with those of Dimeo et al. (31), who conducted an 8- to 12-week treadmill-based intervention and observed significant improvements in the effectiveness of aerobic activity after 8 weeks. Similarly, Cao et al. (35) demonstrated that aerobic activity of less than 8 weeks and more than 12 weeks was similarly effective in decreasing blood pressure. In another study, John et al. (33) portrayed a significant reduction in systolic blood pressure in the training group (−3.8 + 2.8 mmHg) and a reduction in diastolic blood pressure (−2.9 + 2.2 mmHg).

The substantial heterogeneity (*I^2^* = 78%) found is common in similar studies due to differences in intervention duration, exercise intensity, and participant characteristics. Thus, the results highlight the importance of aerobic activity as a non-pharmacological intervention in the reduction of blood pressure among adults. The stronger effects observed in shorter follow-up periods (≤ 8 weeks) align with findings demonstrating that individual responses from patients with uncontrolled BP showed a reduction in 24-h systolic BP (from ~ 2 to ~ 6 mmHg) after 8 weeks of training (34). This observation reflects that initial blood pressure reductions can be more pronounced but may stabilize or diminish over time if exercise intensity is not maintained. This highlights the importance of re-evaluations to maintain the intensity for a long time with sustained interventions for long-term blood pressure management (31).

In conclusion, adherence to antihypertensive medication together with aerobic activity effectively reduced blood pressure among hypertensive adults in Africa, with the most notable effect at 8 weeks and a slightly diminished effect in prolonged weeks, which calls for more research on sustained outcomes. The results highlight the urgent need for integrating structured aerobic activity programs into blood pressure management protocols at local healthcare practices. Promoting aerobic activity as a cost-effective, non-pharmacological intervention could be highly impactful in blood pressure reduction. In addition, most of the studies presented in this review and meta-analysis reported significant heterogeneity in blood pressure reduction. This finding calls for future research to explore the factors for such variations, such as genetic differences, lifestyle factors, and adherence to exercise protocols. Concerning future research, the current evidence is limited by the underrepresentation of women, hence calling for more interventional studies to involve all genders in the evaluation of interventional outcomes through non-pharmacological measures. Concerning timing and duration, since the effects appear to be stronger for a follow-up time of 8 weeks, authorities should promote interventions that focus on early monitoring and intensive treatment or management within shorter periods. Moreover, in this study, we hypothesize that the diminishing effect of aerobic activity over time may be due to physiological adaptations and individual factors such as age, fitness level, genetics, or inconsistencies in exercise activities over a long period. These inconsistencies could jeopardize the initial observed benefits, leading to a lack of sustainability in physical exercises in the long run. This study highly recommends further investigation into why the effect diminishes after periods longer than 8 weeks.

This study has several limitations. One notable issue is the underrepresentation of women, with only one study (27) including women in the intervention, leading to a lack of generalizability to the broader population (both women and men). Additionally, the studies included in this review used Omron digital machines to measure blood pressure. Due to varying machine models, there may be a possibility of lacking precision in blood pressure readings, which makes it difficult to draw consistent conclusions. High heterogeneity was persistent as the studies used different forms of aerobic physical activities, durations, and intensities. Publication bias and small study effects might account for the effects we observed in the results as well as the varying estimates in both SBP and DBP. However, due to the limited number of studies included in the meta-analysis (only five), we could not plot a funnel plot to visualize publication bias. Furthermore, due to the small number of studies included in this study, the interpretation of this study’s findings should be approached with caution, as the generalizability of the results is biased toward the male population across African regions. Future studies should address these limitations to obtain more generalizable findings by including female populations and measuring blood pressure in line with WHO recommendations, incorporating various forms of aerobic physical activities.

## Data Availability

The original contributions presented in the study are included in the article/supplementary material, further inquiries can be directed to the corresponding author.
